# Impact of Tocopherol Supplementation on Clinical Parameters of Periodontal Disease: A Systematic Review and Meta-Analysis

**DOI:** 10.3390/jpm14101039

**Published:** 2024-09-28

**Authors:** Bogdan Andrei Bumbu, Magda Mihaela Luca, Roxana Buzatu

**Affiliations:** 1 Department of Dental Medicine, Faculty of Medicine and Pharmacy, University of Oradea, 410073 Oradea, Romania; bogdanbumbu@uoradea.ro; 2 Department of Pediatric Dentistry, Faculty of Dental Medicine, “Victor Babes” University of Medicine and Pharmacy Timisoara, Eftimie Murgu Square 2, 300041 Timisoara, Romania; 3 Department of Dental Aesthetics, Faculty of Dental Medicine, “Victor Babes” University of Medicine and Pharmacy Timisoara, Revolutiei Boulevard 9, 300041 Timisoara, Romania; roxana.buzatu@umft.ro

**Keywords:** periodontitis, dentistry, tocopherol, periodontal disease, vitamin E

## Abstract

**Background and Objectives**: The significance of periodontal disease as a public health issue prompts the exploration of effective treatments, including the potential use of tocopherol (Vitamin E) due to its anti-inflammatory and antioxidant properties. **Materials and Methods**: The PICO statement (Population, Intervention, Comparator, Outcome) was as follows: In patients with periodontal disease, does tocopherol (Vitamin E) supplementation compared to no supplementation or insufficient Vitamin E intake improve clinical outcomes such as gingival inflammation, pocket depth, and clinical attachment levels? This study searched through PubMed, Scopus, and Web of Science up to June 2024 focused on studies involving human subjects with various forms of periodontal disease, analyzing the impact of tocopherol through dietary or supplementary intake. Primary outcomes evaluated included improvements in gingival inflammation, pocket depth, and clinical attachment levels, with data synthesis conducted according to PRISMA (Preferred Reporting Items for Systematic Reviews and Meta-Analyses) guidelines. Quality assessment and risk of bias were meticulously performed for the included observational studies and randomized controlled trials. **Results**: The meta-analysis incorporated 8 studies that were used for data extraction, totaling 12,832 patients, revealing a heterogeneous response to tocopherol supplementation, with a pooled odds ratio for efficacy in reducing periodontal disease severity at about 0.97 (95% CI: 0.96–0.98). Noteworthy findings indicated a statistically significant increase in clinical attachment loss and pocket depth with odds ratios ranging from 1.15 to 9.33 when Vitamin E was insufficient. However, the considerable heterogeneity (I^2^ = 88.35%) underscores variations in tocopherol’s effectiveness across different populations and study designs. **Conclusions**: While tocopherol supplementation shows a modest benefit in managing periodontal disease, particularly in reducing clinical attachment levels and pocket depth, the variability in outcomes emphasizes the necessity for more research to establish standardized treatment protocols and dosages.

## 1. Introduction

Periodontal disease, encompassing a range of inflammatory conditions affecting the supporting structures of the teeth, represents a significant global health burden. It is estimated that severe periodontal disease affects nearly 10% of the global population, making it the sixth most prevalent health condition worldwide [1,2,3]. The multifactorial etiology of periodontal disease includes bacterial plaque as the primary causative agent, modulated by host and environmental factors. Chronic periodontitis, the most common form of the disease, leads to the progressive loss of the alveolar bone around the teeth, which is a major cause of tooth loss in adults [4,5,6].

Recent epidemiological data highlight the rising incidence of periodontal disease, exacerbated by increasing life expectancies and changes in lifestyle factors that influence oral health, such as smoking and diet [7,8]. The link between periodontal disease and systemic health issues, including cardiovascular disease, diabetes, and respiratory conditions, underscores the importance of effective management strategies for periodontal health [9,10,11]. Additionally, emerging research indicates a possible connection between periodontal disease and neurodegenerative conditions, suggesting that the inflammation originating in the mouth may have far-reaching effects on overall health [12].

The role of antioxidants in managing periodontal disease has garnered attention due to their potential to modulate the oxidative stress associated with the inflammatory processes [13]. Tocopherol, commonly known as Vitamin E, is a lipid-soluble antioxidant that is known to counteract oxidative stress by neutralizing free radicals [14,15]. Several in vitro and in vivo studies suggest that tocopherol can influence inflammatory markers and potentially improve clinical outcomes in patients with periodontal disease [16,17]. However, the extent and nature of its efficacy remain underexplored in comprehensive clinical settings.

Other recent studies indicate an increasing number of studies focusing on the adjunctive role of antioxidants such as Vitamin E in periodontal therapy [18]. Nonetheless, these studies vary significantly in their design, methodology, and outcomes, which complicates the aggregation of data and the formulation of definitive conclusions. Meta-analyses in this field have also been limited and have reported mixed results, emphasizing the need for a rigorous evaluation of existing research to ascertain the effectiveness of tocopherol as a therapeutic adjunct in the treatment of periodontal disease [18].

The primary objective of this systematic review is to assess the efficacy of tocopherol in reducing the clinical severity of periodontal disease, as evidenced by improvements in gingival index, pocket depth, and clinical attachment levels. The hypothesis driving this research is that tocopherol supplementation, when used as an adjunct to conventional periodontal therapy, provides a statistically significant improvement in clinical outcomes. This systematic review and meta-analysis aim to provide a comprehensive assessment of the available evidence, offering insights into the potential integration of tocopherol into standard periodontal treatment regimens.

## 2. Materials and Methods

### 2.1. Eligibility Criteria and Information Sources

The literature search was conducted using the databases PubMed, Scopus, and Web of Science, with the search being limited to studies published up until June 2024.

The PICO statement for this study was as follows: In patients with periodontal disease—including gingivitis and periodontitis—does supplementation with tocopherol (Vitamin E), either through dietary intake or supplements (compared to no supplementation or insufficient Vitamin E intake), improve clinical outcomes such as reduced gingival inflammation, decreased pocket depth, enhanced clinical attachment levels, and reduce periodontal disease severity as indicated by statistical measures like pooled odds ratios?

The inclusion criteria encompassed studies involving human participants diagnosed with periodontal diseases like gingivitis and periodontitis; research specifically investigating the effects of tocopherol through dietary intake or supplementation; studies reporting on periodontal health outcomes such as the reduction in gingival inflammation, decreased pocket depth, or improvements in clinical attachment levels; randomized controlled trials, observational studies (cohort, case-control, cross-sectional), and other relevant clinical studies; and peer-reviewed articles published only in English.

The exclusion criteria included studies focusing on multivitamin supplements or broader dietary patterns that do not isolate the effects of tocopherol; research that does not report on the specific periodontal health outcomes of interest; animal studies, in vitro studies, and ex vivo studies; studies lacking clear, quantifiable data on periodontal outcomes or those that do not provide sufficient detail for a thorough analysis; grey literature such as preprints, conference proceedings, dissertations, and other non-peer-reviewed publications; and studies that are duplicate reports or extensions without new relevant data. Additionally, reviews were not considered for inclusion in this study.

### 2.2. Search Strategy

Our search strategy for this systematic review and meta-analysis utilized a carefully selected array of keywords and Medical Subject Heading (MeSH) terms to capture studies pertinent to the role of tocopherol in periodontal disease management. The keywords and MeSH terms used were as follows: “Vitamin E”, “tocopherol”, “antioxidants”, “periodontal health”, “periodontitis”, “gingivitis”, “periodontal disease”, “dental health”, “gingival health”, “oral health”, “alveolar bone loss”, “dental plaque”, “immune response”, “inflammatory response”, “oxidative stress”, “clinical outcomes”, and “treatment efficacy”.

To effectively structure the search, Boolean operators were employed to combine these terms in a way that would refine and focus the search on the most relevant literature. The constructed search string was as follows: (((“Vitamin E” OR “tocopherol”) AND (“antioxidants”) AND (“periodontal health” OR “periodontitis” OR “gingivitis” OR “periodontal disease” OR “dental health” OR “gingival health” OR “oral health”) AND (“immune response” OR “inflammatory response” OR “oxidative stress”) AND (“clinical outcomes” OR “treatment efficacy” OR “disease progression” OR “alveolar bone loss”))).

### 2.3. Data Collection and Selection Process

Following the Preferred Reporting Items for Systematic Reviews and Meta-Analyses (PRISMA) guidelines [19], our data collection and selection process were designed to ensure accuracy, reproducibility, and transparency in our systematic review and meta-analysis focusing on the role of tocopherol in periodontal disease. Initially, all retrieved records from the executed comprehensive database searches were independently screened by two reviewers. During this preliminary phase, each record was assessed based on our predefined inclusion and exclusion criteria to determine its eligibility for inclusion in our study.

In cases where discrepancies arose between reviewers during this initial screening phase, the records in question were discussed to reach a consensus. If agreement could not be achieved through discussion, the record was escalated to a third reviewer for a final decision. To enhance the efficiency of this process and reduce the potential for errors, we employed automated tools to manage and track the screening process. This system aided in organizing the records, eliminating duplicates, and ensuring a streamlined progression to the subsequent stages of abstract and full-text review.

Each study that passed the initial screening was then subjected to a more detailed evaluation, where both abstracts and full texts were reviewed to confirm their relevance and adherence to the inclusion criteria. The entire selection process was meticulously documented and has been registered on the Open Science Framework (OSF). The registration code and relevant details can be accessed at osf.io/fb6wh.

### 2.4. Data Items

For this systematic review, data were manually extracted from the selected studies. The primary data points of interest were key clinical indicators of periodontal health, including changes in the gingival index, pocket depth, and clinical attachment levels as a result of tocopherol intake or supplementation. Secondary data collected encompassed participant demographics such as age, gender, smoking status, and the presence or absence of diabetes, which are crucial for understanding the contextual factors that might influence periodontal outcomes. Study-specific information, including the country of origin, year of publication, study design, and sample size, was also meticulously collected to evaluate the robustness and generalizability of the findings. Moreover, to ensure the reliability and applicability of the results, the quality of each study was assessed, and outcomes were aligned with standardized clinical measures often employed in periodontal research, such as periodontal pocket depth measurements and clinical attachment loss evaluations.

### 2.5. Risk of Bias and Quality Assessment

To evaluate the risk of bias and the quality of studies included in our systematic review and meta-analysis of tocopherol’s role in periodontal disease management, we employed a dual approach tailored to the study designs. For observational studies, we used the Newcastle–Ottawa Scale [20]. This scale is a recognized tool that assesses three critical aspects: the selection of study groups, the comparability of the groups, and the ascertainment of the exposure or outcomes. Studies are awarded stars in these categories, culminating in an overall quality score that classifies each study as low, medium, or high quality.

For randomized controlled trials (RCTs), we applied the Cochrane Collaboration’s tool for assessing the risk of bias. This tool evaluates several key domains: random sequence generation, allocation concealment, blinding of participants and personnel, blinding of outcome assessment, completeness of outcome data, selective reporting, and other potential biases. Each domain is rated as having a ‘low risk’, ‘high risk’, or ‘unclear risk’ of bias. Two independent researchers performed these assessments (B.A.B. and R.B.), with any discrepancies resolved through discussion or, if necessary, consultation with a third reviewer (M.M.L.).

### 2.6. Synthesis Methods

Our synthesis methods for evaluating the impact of tocopherol (Vitamin E) on periodontal health combined both qualitative and quantitative approaches. We included studies that provided specific data on tocopherol intake and its clinical effects on periodontal outcomes such as pocket depth reduction, gingival inflammation, and clinical attachment level improvement. Data were systematically organized in tables, highlighting outcomes related to periodontal health improvements and levels of tocopherol intake. Any missing data were identified, and their potential impacts on the study findings were critically evaluated.

A meta-analysis was conducted to quantify the effectiveness of tocopherol supplementation or dietary intake on specific periodontal health outcomes. We assessed heterogeneity among the study results using the I^2^ statistic, which indicates the percentage of variation across studies due to heterogeneity rather than chance. High I^2^ values suggest significant variability, essential for interpreting the efficacy of tocopherol in periodontal disease management. All statistical analyses were performed using Python statistical software (v.3.7.16), and the results were presented with pooled odds ratios and 95% confidence intervals to provide precise estimates of the observed effects.

## 3. Results

### 3.1. Study Selection and Study Characteristics

**Figure 1:**
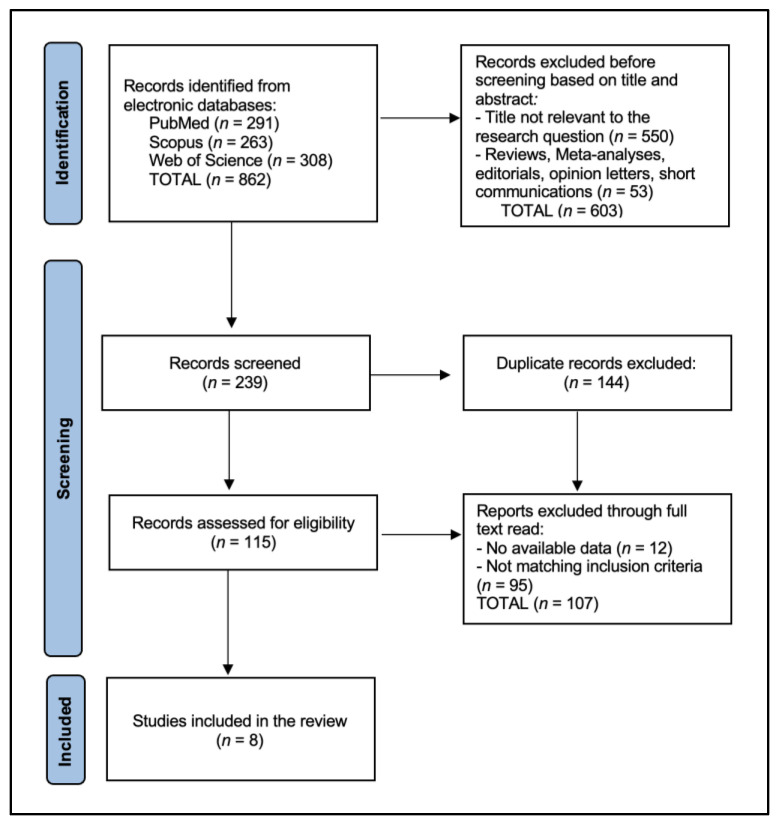
PRISMA Flow Diagram.

The initial search identified 862 articles. After removing 144 duplicate entries, 603 records were excluded based on their titles and abstracts before screening. Following a full review, 95 articles were excluded for not meeting the inclusion criteria or lacking available data. Ultimately, the systematic review included 8 eligible studies in the final analysis, as shown in Figure 1 and Table 1 [21,22,23,24,25,26,27,28].

### 3.2. Results of Individual Studies

Table 2 provides a detailed overview of the patient backgrounds across eight distinct studies that evaluated the effects of periodontal disease. This analysis involved a comprehensive examination of 12,832 patients who were divided into various groups based on the severity of their periodontal conditions and compared to 31,232 individuals without the disease. Age and gender distribution varied considerably among these groups. For instance, patients with mild to severe disease conditions ranged in age from 20.3 years in the youngest subgroup analyzed by Hosoda et al. [24] to 70 years in the oldest subgroup studied by Iwasaki et al. [28]. The percentage of male participants fluctuated across these studies, from none reported by Hosoda et al. [24] to 70.4% in those with severe disease in the study by Luo et al. [23].

### 3.3. Results of Synthesis

Chapple et al. [21] reported only a slight protective effect of adequate Vitamin E levels, with an odds ratio (OR) of 0.97 (95% CI: 0.90–1.05), suggesting that while sufficient Vitamin E may help, its impact is relatively modest. Li et al. [22] observed a similar trend with an OR of 0.96 (95% CI: 0.95–0.98), indicating minimal but consistent protective benefits from adequate vitamin intake.

Contrastingly, Luo et al. [23] noted a significantly increased risk associated with insufficient Vitamin E, with an OR of 1.57 (95% CI: 1.22–2.03), emphasizing the potentially detrimental effects of vitamin deficiencies on periodontal health. The most dramatic findings came from Behfarnia et al. [27], where insufficient Vitamin E was linked to a dramatically higher risk of periodontal disease, marked by an OR of 9.33 (95% CI: 4.87–17.89), highlighting a severe impact of nutritional deficit.

Interestingly, the studies varied significantly in their inclusion of participants with complicating factors such as smoking and diabetes. For example, Hosoda et al. [24] and Behfarnia et al. [27] excluded participants with these conditions, potentially skewing results towards a less compromised population and resulting in a very low OR of 0.52 (95% CI: 0.28–0.98) in Hosoda’s study. In contrast, studies like Zong et al. [26] included participants with a high prevalence of diabetes (36%), which could influence the disease’s progression and complicate the assessment of Vitamin E’s impact, as reflected in their higher OR of 1.65 (95% CI: 1.26–2.16), as seen in Table 3.

**Figure 2:**
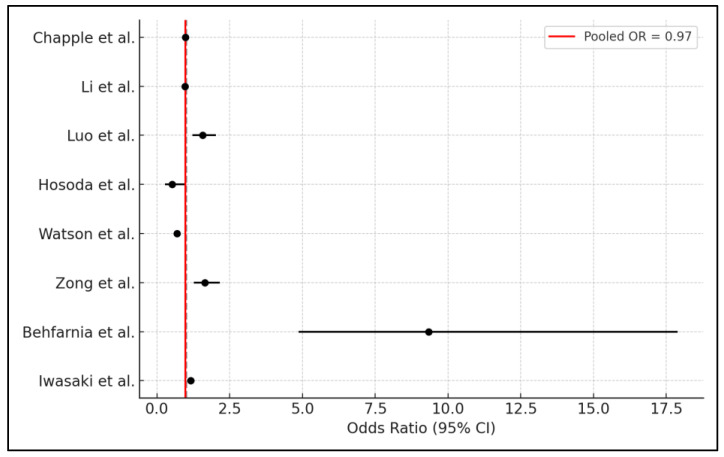
Forest plot of meta-analysis [21,22,23,24,25,26,27,28].

In the meta-analysis examining the effects of periodontal disease, the weights assigned to each study, reflecting their relative contribution based on the total number of participants, are as follows: Chapple et al. [21] accounts for the largest share at 43.42%, followed by Watson et al. [25] at 17.92%, and Li et al. [22] at 16.94%. Luo et al. [23] contributes 12.13%, while Zong et al. [26] adds 8.90%. The smaller studies, Iwasaki et al. [28], Hosoda et al. [24], and Behfarnia et al. [27], contribute 0.42%, 0.23%, and 0.03%, respectively, indicating a lesser influence in the overall analysis due to their smaller sample sizes. The meta-analysis resulted in a pooled odds ratio of approximately 0.97, with a 95% confidence interval ranging from 0.96 to 0.98. This suggests a marginally significant association between higher Vitamin E intake and improved periodontal outcomes, indicating that Vitamin E may confer a slight protective effect against periodontal disease. However, the high heterogeneity among the included studies is indicated by an I^2^ value of 88.35%, as presented in Figure 2.

## 4. Discussion

### 4.1. Assessment of Findings and Additional Literature

Based on the collective findings from eight studies involving 12,832 patients, maintaining sufficient Vitamin E levels appears to have a modest protective effect on periodontal health, with a pooled odds ratio of 0.97 (95% CI: 0.96–0.98), indicating a slight reduction in disease severity. However, only one study (Behfarnia et al. [27]) specified an exact dosage, where patients taking 200 International Units of Vitamin E daily for two months showed significant improvements in periodontal status, reflected by a high odds ratio of 9.33 (95% CI: 4.87–17.89). The other studies assessed Vitamin E intake through dietary evaluations or serum levels without providing specific dosages. Due to the considerable variability among studies (I^2^ = 88.35%) and the lack of consistent dosing information, it is not possible to determine an exact recommended dose from the overall data; however, the 200 IU daily dosage from the Behfarnia et al. [27] study [27] may serve as a reference point until more standardized dosing guidelines are established through further research.

The methodological differences across the studies, particularly in the assessment of periodontal health metrics such as clinical attachment level and pocket depth, add another layer of complexity to interpreting the data. These discrepancies in the diagnostic criteria and outcome measurements may account for the high heterogeneity observed in the meta-analysis results. The inconsistency in results underscores the need for standardization in clinical trials investigating tocopherol’s effects on periodontal outcomes.

Despite some studies reporting minimal benefits, the overall synthesis of data suggests that tocopherol has a place in the adjunctive treatment of periodontal disease, particularly in populations at risk of dietary deficiency. The evidence points towards a nuanced understanding of tocopherol’s role, suggesting that while it is not a panacea, it holds potential value in comprehensive periodontal therapy, especially when considering individual patient dietary backgrounds and systemic health conditions.

In a similar manner, the review by Mi et al. [18] demonstrated that the higher dietary intake of various vitamins, including Vitamin E, was significantly associated with a reduced risk of periodontal disease, presenting odds ratios that underscored the protective effects of vitamins (e.g., Vitamin E OR: 0.868, 95% CI: 0.776–0.971). This finding aligns with the results presented by Nizam et al. [29], who investigated the impact of alpha-tocopherol, a form of Vitamin E, along with selenium on gingival and periodontal ligament fibroblasts in vitro. Their research showed that alpha-tocopherol alone and in combination with selenium not only enhanced the proliferation and healing rates of these cells but also increased the release of basic fibroblast growth factor and collagen type I over several time points. Specifically, the combination treatment significantly increased the healing rates of periodontal ligament fibroblasts at 12 and 48 h, indicating a potent synergistic effect that could translate into clinical benefits in the management of periodontal disease.

Similarly, the study by Shadisvaaran et al. [16] reviewed the preclinical and clinical impacts of Vitamin E on periodontitis and suggested that Vitamin E could enhance periodontal health by reducing oxidative stress and inflammatory responses, as well as by promoting wound healing. Despite these potential benefits, they noted that direct evidence from human clinical trials remains scarce and called for more rigorous studies to confirm these findings. Correspondingly, Dodington et al. [30] found that higher intakes of fruits and vegetables, as well as specific nutrients like β-carotene, Vitamin C, and α-tocopherol, were positively associated with periodontal healing post-nonsurgical therapy in nonsmokers. They reported significant reductions in probing depth with an increased intake of these antioxidants (*p* < 0.05), highlighting the role of a nutrient-rich diet in enhancing periodontal recovery. However, they noted no significant benefits in smokers, underscoring the possible interference of smoking with the healing processes mediated by these nutrients. Both studies underscore the potential of Vitamin E and other antioxidants in periodontal therapy, yet also emphasize the variability in outcomes based on individual factors such as smoking.

Additionally, the study by Matthews-Brzozowska et al. [31] found significant improvements in periodontal health following the oral application of Coenzyme Q10 with Vitamin E in patients with chronic periodontitis. Over a two-month period, notable reductions were observed in plaque index (PII) from 1.0 to 0.36, and the interdental hygiene index (HYG) from 39.51% to 6.97%. Additionally, the gingival index (GI) decreased from 0.68 to 0.18, and the sulcus bleeding index (SBI) dropped dramatically from 7.26 to 0.87. These clinical improvements were complemented by a 30% reduction in periodontal pocket depth and a 20% improvement in the total antioxidant status in mixed saliva. Similarly, the study by Zhang et al. [32] explored the relationship between alpha-tocopherol intake and cognitive performance, highlighting the mediating role of periodontitis. They reported that higher intakes of alpha-tocopherol were significantly associated with better cognitive performance scores such as CERAD, AFT, and DSST, with odds ratios indicating substantial reductions in the risk of cognitive decline for the highest versus lowest tertile of intake (ORs ranged from 0.214 to 0.298). Moreover, clinical periodontitis appeared to adversely affect cognitive scores, suggesting that periodontal health could be a critical mediator in the link between nutrient intake and cognitive function.

The clinical utility of tocopherol in the management of periodontal disease, as evidenced by the systematic review and meta-analysis, emphasizes its potential role in enhancing standard periodontal treatment regimens. The improvement observed in gingival index, pocket depth, and clinical attachment levels in patients receiving tocopherol supplementation suggests that this antioxidant can significantly aid in the reduction of inflammation and the stabilization of periodontal health. This could be particularly beneficial in patients who exhibit poor response to conventional therapies or those with exacerbated oxidative stress profiles, common in chronic periodontitis. By potentially modifying the disease’s clinical trajectory, tocopherol supplementation offers a promising adjunctive therapy that can be easily integrated into existing periodontal care protocols, providing a non-invasive option to help manage and mitigate periodontal disease progression.

### 4.2. Study Limitations

This review is not without limitations. The exclusion of grey literature and the high variability in the quality of included studies may impact the generalizability of the findings. Furthermore, the significant heterogeneity noted (I^2^ = 88.35%) indicates substantial differences in study designs, participant selection, and tocopherol dosages, which complicates the direct comparison of results. Nevertheless, controlling for potential confounders was not possible due to the study design. These factors highlight the need for a cautious interpretation of the data and the consideration of underlying biases that might influence the outcomes.

## 5. Conclusions

In conclusion, this systematic review indicates that tocopherol supplementation could play a beneficial role in the management of periodontal disease, albeit with modest effects. The contrasting results across studies highlight the importance of maintaining adequate tocopherol levels to prevent severe periodontal outcomes. However, given the variability in study results and methodologies, further research is needed to conclusively determine the optimal usage and dosing of tocopherol in different patient populations. Healthcare providers should consider individual patient profiles and existing comorbidities when recommending tocopherol as part of periodontal therapy to enhance clinical outcomes effectively.

## Figures and Tables

**Table 1:** Characteristics of the studies included in the review.

Study and Author	Country	Study Year	Study Design	Quality Assessment
1 [21] Chapple et al.	USA	2007	Cross-sectional	Medium
2 [22] Li et al.	China	2022	Cross-sectional	High
3 [23] Luo et al.	China	2018	Cross-sectional	Medium
4 [24] Hosoda et al.	Japan	2021	Cross-sectional	Medium
5 [25] Watson et al.	United Kingdom	2022	Cross-sectional	High
6 [26] Zong et al.	United Kingdom	2015	Retrospective cohort	High
7 [27] Behfarnia et al.	Iran	2021	Clinical trial	High
8 [28] Iwasaki et al.	Japan	2011	Retrospective cohort	Medium

**Table 2:** Patients’ background characteristics.

Study and Author	Number of Participants	Comparison Group	Age (Category/Mean/Median), Years	Gender (Male)
1 [21] Chapple et al.	Mild disease: 1567 patients Severe disease: 609 patients	No periodontal disease: 20,784	Mild disease: 52.2 Severe disease: 56.4	Mild disease: 61.1% Severe disease: 68.1%
2 [22] Li et al.	Moderate/Severe periodontitis: 3994 patients	No periodontal disease: 4965	Mean: 56.7	57.9%
3 [23] Luo et al.	Moderate disease: 2274 patients Severe disease: 676 patients	No periodontal disease: 3465	Moderate disease: 55.3 Severe disease: 54.5	Moderate disease: 54.1% Severe disease: 70.4%
4 [24] Hosoda et al.	PD: 49 patients	Non-PD: 71 patients	PD: 20.3 Non-PD: 20.4	0.0%
5 [25] Watson et al.	High-risk of PD: 1634 patients	Low-risk of PD: 7842 patients	High-risk for PD: 54.7 Low-risk for PD: 56.4	High-risk for PD: 36.3% Low-risk for PD: 44.1%
6 [26] Zong et al.	PD: 4708 patients	Quartiles of tocopherol serum levels	Mean: 45.7	48.4%
7 [27] Behfarnia et al.	Vitamin E supplementation: 7 patients (cases)	No supplementation: 7 patients (controls)	Cases: 44.4 Controls: 40.7	NR
8 [28] Iwasaki et al.	224 patients	Tertiles of tocopherol serum levels	70 years	48.2%

PD—Periodontal Disease; NR—Not Reported.

**Table 3:** Evaluation of the risk of periodontal disease development.

Study and Author	Periodontal Disease Assessment	Smoking Status and Diabetes Status	Vitamin E Assessment	Risk Assessment (OR/HR/RR—95% CI)
1 [21] Chapple et al.	At least one site with both clinical attachment loss ≥4 mm and probing pocket depth of ≥4 mm	Mild disease (smoking): 25.8% Severe disease (smoking): 21.4% Mild disease (diabetes): 12.2% Severe disease (diabetes): 15.3%	Quintile 1: 16.42 μmol/L Quintile 2: 20.06 μmol/L Quintile 3: 23.45 μmol/L Quintile 4: 27.98 μmol/L Quintile 5: 37/48 μmol/L	Sufficient Vitamin E: OR = 0.97 (0.90–1.05)
2 [22] Li et al.	≥2 Interproximal sites with a clinical attachment loss (CAL) of ≥4 mm; ≥2 Interproximal sites with a periodontal probing depth of ≥5 mm	Smoking: 17.8% Diabetes: 21.2%	Insufficient Vitamin E: 91.2% with mild and moderate periodontitis	Sufficient Vitamin E: OR = 0.96 (0.95–0.98)
3 [23] Luo et al.	At least two interproximal sites with PD of at least 5 mm not occurring on the same tooth, or at least two interproximal sites which are not on the same tooth, and which have an AL of at least 4 mm.	Moderate disease (smoking): 29.8% Severe disease (smoking): 27.4% Moderate disease (diabetes): 15.4% Severe disease (diabetes): 13.6%	Severe periodontal disease: Quartile 1 <4.55 mg: 29.5% Quartile 2 4.55–7.09 mg: 23.7% Quartile 3 7.10–11.10 mg: 2.8% Quartile 4 > 11.11 mg: 24.0%	Insufficient Vitamin E: OR = 1.57 (1.22–2.03)
4 [24] Hosoda et al.	Code 0: healthy periodontal conditions; Code 1: gingival bleeding on probing; Code 2: Calculus and bleeding; Code 3: Periodonal pocket 4–5 mm; Code 5: periodontal pocket >6 mm	Smoking: 0.0% Diabetes: 0.0%	PD: 144 μg/1000 kcal Non-PD: 167 μg/1000 kcal	Sufficient Vitamin E: OR = 0.52 (0.28–0.98)
5 [25] Watson et al.	Self-reported diagnosis of periodontal disease	Smoking: 6.6% Diabetes: NR	NR	Sufficient Vitamin E: OR = 0.69
6 [26] Zong et al.	Mean CAL, mean PPD, and periodontitis were calculated using data collected at mesiobuccal sites for consistency between survey circles, and interproximal sites are more reflective of periodontitis than midbuccal sites. Total periodontitis (TPD) was defined as the sum of mild, moderate, and severe periodontitis according to CDC	Smoking: 18.0% Diabetes: 36.0%	Mean levels: 30.7 μmol/L Quartile 1: 20.2 μmol/L; Quartile 2: 24.5 μmol/L; Quartile 3: 29.7 μmol/L; Quartile 4: 48.2 μmol/L	Insufficient Vitamin E (quartile 1 with an average serum tocopherol of 20.2 μmol/L): OR = 1.65 (1.26–2.16)
7 [27] Behfarnia et al.	The case group received 200 IU supplementary Vitamin E (E-Vigel 200, Dana Pharma Co, Iran) to consume daily up to 2 months. The amount of clinical attachment loss (CAL) between 1 and 5 mm.	Smoking: 0.0% Diabetes: 0.0%	Case group received 200 IU supplementary Vitamin E	Insufficient Vitamin E: OR = 9.33 (4.87–17.89)
8 [28] Iwasaki et al.	The periodontal condition, measured as CAL (mean CAL at baseline = 3.1). A loss of attachment of 3 mm or greater in 1 year at any site was considered periodontal disease progression	Smoking: 12.5% Diabetes: 6.3%	Mean levels: 12.5 μg/mL	Insufficient Vitamin E (lowest tertile): OR = 1.15 (1.04–1.28)

NR—Not Reported; OR—Odds Ratio; RR—Risk Ratio; PD—Periodontal Disease; PD—Pocket Depth; CAL—Clinical Attachment Loss.

## Data Availability

Not applicable.
